# Immune Reconstitution in the Aging Host: Opportunities for Mechanism-Based Therapy in Allogeneic Hematopoietic Cell Transplantation

**DOI:** 10.3389/fimmu.2021.674093

**Published:** 2021-04-19

**Authors:** Richard J. Lin, Harold K. Elias, Marcel R. M. van den Brink

**Affiliations:** ^1^Adult Bone Marrow Transplantation (BMT) Service, Division of Hematologic Malignancies, Memorial Sloan Kettering Cancer Center, New York, NY, United States; ^2^Department of Medicine, Weill Cornell Medical College, New York, NY, United States

**Keywords:** aging, cellular senescence, mitochondrial dysfunction, immune reconstitution, allogeneic hematologic stem cell transplantation

## Abstract

Older patients with hematologic malignancies are increasingly considered for allogeneic hematopoietic cell transplantation with encouraging outcomes. While aging-related thymic dysfunction remains a major obstacle to optimal and timely immune reconstitution post- transplantation, recent accumulating evidence has suggested that various aging hallmarks such as cellular senescence, inflamm-aging, and hematopoietic stem cell exhaustion, could also impact immune reconstitution post-transplantation in both thymic-dependent and independent manner. Here we review molecular and cellular aspects of immune senescence and immune rejuvenation related to allogeneic hematopoietic cell transplantation among older patients and discuss potential strategies for mechanism-based therapeutic intervention.

## Introduction

In recent years, allogeneic hematopoietic cell transplantation (HCT), the original form of cellular immunotherapy, is increasingly utilized in older adults (1). This trend is likely due to the increasing incidence of hematologic malignancies in older adults; improved toxicity profiles of conditioning regimen, novel graft manipulation and graft-versus-host disease (GVHD) prevention strategies; as well as better selection, optimization, and supportive care of the geriatric patient (1–3). Importantly, outcomes for older patients in allogeneic HCT are improving gradually and many older patients are now routinely evaluated for and undergo the lifesaving procedure (1, 4–6).

One of the most important aspects of successful allogeneic HCT in older adults is the capacity for donor immune reconstitution, especially the optimal recovery of T-cell adaptive immunity critical for both anti-infection and anti-tumor activities (7, 8). However, in the context of an aging host, delayed immune reconstitution may be caused by age-related thymic atrophy/involution, suppressive inflammatory cytokine milieu, or HCT-associated complications such as infection or GVHD (9, 10). In this mini-review, we discuss immune reconstitution in the context of existing aging hallmarks and pathways, its clinical relevance to transplant outcomes, and opportunities for mechanism-based interventions to improve clinical outcomes for older patients.

## Hallmarks of Aging and the Immune System

Recent development in the field of geroscience has provided a unique opportunity to examine the issue of immune reconstitution following HCT in older adults. The normal healthy aging process is defined by at least nine different and relatively conserved denominators of aging hallmarks including stem cell exhaustion, cellular senescence, mitochondrial dysfunction, epigenetic alterations, telomere attrition, genomic instability, altered intercellular communication, loss of proteostasis, and deregulated nutrient sensing (11). Perturbations or exacerbated responses in any of these pathways are known to cause pathologic states such as cancer, neurodegenerative disease, or cardiovascular disease (11, 12). We highlight here several of these hallmarks which are evident in the human immune system and more importantly, potential druggable targets for intervention to improve outcomes of older patients in allogeneic HCT.

### Stem Cell Aging

There are many cell-intrinsic changes in hematopoietic stem cells (HSCs), innate and adaptive immune cells, as well as microenvironmental changes in the bone marrow, and primary and secondary lymphoid organs during aging (13). The first of which is the gradual age-related functional decline in the hematopoietic compartment, especially of HSCs. Aged HSCs exhibit impaired self-renewal and long-term reconstitution potential both in competitive and noncompetitive settings (14, 15), presumably due to the increased proliferative stress following HSC engraftment (16, 17). Furthermore, accumulating data indicate that decreased lymphoid production in the elderly is in part caused by cell-intrinsic alterations in lineage potential of HSCs and their committed progenitors as evident in numerous transcriptional and epigenetic profiling studies in aged HSCs from mice (17–20) and humans (21). At the molecular level, increased Wnt5a expression in HSCs induces elevated RhoGTPase Cdc42 signaling and alteration of histone H4K16 distribution, thereby mediating the aging phenotype (22, 23). Importantly, pharmacologic inhibition of Cdc42 activity, by Cdc42 activity-specific inhibitor (CASIN), can reverse the aging phenotype and reconstitute a “younger” immune system based on gene expression profiles and DNA vaccine responses in mouse transplantation models (24).

These preclinical studies have had significant implications in allogeneic HCT especially related to the age of donor HSCs. Several large registry studies have invariably demonstrated the positive impact of younger donor age on allo-HCT outcomes across matched unrelated donors and haploidentical donors (25, 26). Mechanistically, HCT from younger donors are associated with improved immune reconstitution post-HCT based on faster kinetics of CD4+ T cell (both naïve and memory), CD8+ T cell, B cell, and NK cell development, which translated to decreased non-relapse mortality and increased disease-free survival in several transplant platforms (27, 28). Interestingly, a recent study demonstrated that in the haploidentical transplantation setting, older donor age was associated with early alloreactivity and reduced numbers of CD45RA+ Treg in the graft and at immune recovery, suggesting an adverse impact of aging-related change in older donor HSCs (29). These clinical observations correlated well with early murine transplantation experiments demonstrating impaired immune reconstitution post-transplantation with older mice as donors (30, 31).

### Cellular Senescence

A second important hallmark is cellular senescence, defined as a state of stable growth arrest once cells are subjected to significant stress and DNA damage. While this is an intrinsic host defensive mechanism to prevent cancer development, these senescent cells also accumulate in different organs and most importantly, secrete pro-inflammatory cytokine and adhesive molecules (32). This senescence-associated secretory phenotype (SASP) creates a pro-inflammatory milieu that is detrimental to the function of many host cells including the immune system (33). This is especially true for the bone marrow niche which is composed of several cell types that play important modulatory roles in both aging and the development of hematologic malignancies (34, 35). In an elegant murine study, Kusumbe et al., demonstrated a causal link between suppression of endothelial NOTCH signaling and the pro-inflammatory bone marrow milieu in aged mice (36). Restoration of NOTCH signaling was sufficient to ameliorate aging defects in the vascular niche essential for HSC maintenance. Moreover, both intrinsic and extrinsic mechanisms of aging are likely operating in the bone marrow niche. Kuribayashi et al. demonstrated that transplantation of aged HSCs in a young BM successfully recapitulated the transcriptional landscape of young HSCs but not their methylation profile, suggesting the presence of both instructive (BM milieu) and permissive (cell-intrinsic) contributors of HSC aging (37). In another study, infusion of young endothelial cells in aged or lethally irradiated mice enhanced HSC reconstitution and hematopoietic recovery by mitigating damage to the BM vascular microenvironment- exemplifying the role of BM microenvironment in HSC aging (38).

Cellular senescence also applies to the aging of T-cells, especially in the context of immune effector function (39). T-cell aging is characterized by the expression of senescence markers such as CD57 and the loss of CD28+, which is distinct from T-cell exhaustion characterized by the expression of checkpoint inhibitors such as PD-1 (40). These senescent T-cells have been associated with chronic viral infections, reduced IL-2 production, increased IL-6 production, and decreased anti-tumor effector activities (41, 42). Delayed T-cell immune reconstitution in older adults most likely results from age-associated thymic functional decline and the aging host environment in the bone marrow as well as other lymphoid organs (43, 44). Functionally, T-cell aging and delayed immune reconstitution affects T-cell immunity and in the context of allogeneic HCT, may affect the ability to defend against various infections and disease relapse.

### Mitochondrial Dysfunction

Related to the aging of HSC and within the emerging field of immunometabolism lies another tenet of tissue aging: mitochondrial dysfunction. Although an association between these two processes has long been recognized (45, 46), the molecular pathways linking these cellular perturbations are poorly elucidated. Studies investigating bioenergetic utilization in aged HSCs have reported enhanced mitochondrial OXPHOS and increased ROS production (47, 48), thereby compromising HSC function. However, it remains unclear if high ROS levels are a trigger or a consequence of HSC dysfunction. Emerging studies have also attributed HSC aging to changes in the BM microenvironment, but the influence of niche factors on metabolic perturbations in HSCs, are yet to be explored. The integrity of mitochondrial DNA (mtDNA) can also influence HSC aging, as exemplified in DNA polymerase gamma (*Polg)* mutant mice deficient in proof-reading capacity, thereby harboring mutations in mtDNA in HSCs and exhibiting features of accelerated aging across numerous tissues including BM (49). Hematopoietic defects include progressive megaloblastic anemia, erythrodysplasia, and impaired *de novo* B and T cell lymphopoiesis, which are features characteristics of myelodysplastic syndromes (50). For further reading and a more comprehensive overview of the role of mitochondria in HSC function, please refer to the following reviews (51, 52).

Activated T cells undergo extensive intracellular metabolic rewiring thereby engaging/co-opting several mitochondrial processes (glycolysis, biogenesis, glutaminolysis, OXPHOS, one-carbon metabolism), to facilitate their high metabolic demand. Aged T cells (naïve CD4+) exhibit impaired mitochondrial adaptation and attenuation of cellular metabolism. Supplementation of glycine which restores one-carbon metabolism, was sufficient to partially rejuvenate aged T cells (53). In a more recent study (54), *Tfam* (encodes mitochondrial transcription factor) deficiency in mouse T cells disrupted mitochondrial integrity and induced inflammaging, resulting in increased senescence and recapitulating premature aging. Treatment with Nicotinamide riboside, a precursor of oxidized nicotinamide adenine dinucleotide (NAD+) and vitamin B3 analog, attenuated systemic inflammation and ameliorated multiple morbidities in mice with *Tfam*-deficient T cells (skeletal wasting, cardiovascular dysfunction, and restored normal physical activity). An emerging area of investigation of immunosenescence is the role of mitokines- mitochondrial stress-induced soluble factors (GDF15, FGF21, and human), which increase with aging and play an adaptive immunometabolic role to arrest the deleterious effects of aging (55). In the context of allo-HCT, it’s conceivable that primary and secondary effects of dysfunctional mitochondria in aged HSCs and lymphoid precursors confer reduced reconstitution potential, thereby delaying post-transplant immune reconstitution.

### Epigenetic Aging and Telomeres

Finally, additional hallmarks of aging such as epigenetic alterations and telomere shortening have been implicated in post-transplant outcomes (56). Recently, several elegant clinical translational studies compared the biologic age of donor with that of the recipient age and showed that there was significant accelerated aging associated with HCT, manifested as a rapid increase of epigenetic age and telomere shortening (57, 58). Using a panel of DNA methylation-based, epigenetic aging biomarkers, Stolzerl et al. compared the biological age of transplant recipients with their donors and found that after a latency period of epigenetic rejuvenation in donor cells, accelerated epigenetic aging accounted for 2.4 years per chronologic year (59). Most importantly, both this study and an earlier study demonstrated that the donor age was the key determinant of DNA methylation-based, epigenetic aging, supporting again the benefit of using younger donors (59, 60). The mechanism of benefits from younger donors may be explained by their longer telomere length as shown in a CIBMTR study of allogeneic HCT recipients of severe aplastic anemia, where longer donor (rather than recipient) telomere length was associated with improved 5-year overall survival (61). The specific impact of epigenetic aging and telomere attrition on post-HCT immune reconstitution, however, has not been examined in these studies.

## Immune Reconstitution Post-Transplant in Older Patients

Clinically speaking, immune reconstitution post allogeneic HCT in older patients follows similar pattern as younger patients, with both thymus-dependent and independent mechanisms (44, 62). However, the aging of the immune system especially the thymus and the bone marrow significantly impact the kinetics of immune reconstitution, resulting in suboptimal clinical outcomes among older patients post allogeneic HCT. For example, the effective reconstitution of adoptive T-cell immunity, especially a diverse CD4+ T-cell repertoire, requires a functional thymus (63). This is crucial in aging patients where thymic atrophy due to involution is common, resulting in impaired recovery and increased risks of infection among older patients. In contrast, CD8+ T-cells predominantly expand in the periphery thus may be less sensitive to the effect of aging (44, 62). Interestingly, a recent large-scale genome-wide association study has suggested that besides age and gender, a common variant (rs2204985) within the T cell receptor TCRA-TCRD locus is associated with increased thymopoiesis and T cell diversity in transplantation models (64). Moreover, infection with many human herpesviruses especially cytomegalovirus (CMV) has significant impact on the pattern and repertoire of overall T cell and CMV-specific immunity post-allogeneic HCT (65, 66). For further details of thymus-dependent mechanism underlying immune reconstitution and various enhancement strategies, the readers are referred to an excellent recent review (44).

Thymus-independent, peripheral T-cell expansion predominates at least during the first year when mature T cells from the graft are triggered to expand either *via* stimulation by host allo-antigens or through homeostatic proliferation in response to lymphopenia (62). As soon as a graft is infused into a recipient, mature T cells from the graft will encounter and respond to host allo-antigens, such as incompatible major and minor histocompatibility leukocyte antigens, as well as tumor-associated and tumor-specific antigens, which results in both GVHD and graft-versus-tumor (GvT) effect (62). However, the T-cell repertoire generated through a thymic-independent mechanism is limited in diversity and skewed (67). The effect of older age and the SASP environment on thymus-independent immune reconstitution post-HCT has not been specifically studied. However, some studies of early T-cell senescence post-HCT have suggested a potential modulatory role in immune reconstitution. For example, T lymphocytes express p16INK4a exponentially with increasing age, a tumor suppressor involved in cell cycle regulation. Wood et al. measured the expression of p16INK4a in hematopoietic stem cell–derived T cells as well as their cellular functioning both before and after allo-HCT. After transplantation, allogeneic HCT recipients demonstrated a significant increase (1.9-fold) in p16INK4a expression, equivalent to a decade of aging. Importantly, RNA sequencing of T cells both before and after HCT revealed changes in T cell subsets mimicking the changes that occur with normal aging (68).

The type of conditioning regimen and graft resource also play major role in immune reconstitution especially in older adults. Total body irradiation (TBI) is particularly damaging to the thymus in experimental models, and its use in older patients has been associated with increased mortality and treatment-related complications (7). Likewise, different graft sources are associated with different kinetics of immune reconstitution which leads to differential impact on relapse and non-relapsed mortality (69, 70). Both studies demonstrated that allografts from peripheral blood have faster overall kinetics of immune reconstitution than either bone marrow or cord blood, and that faster immune reconstitution of T-cells is associated with better survival outcomes. In addition, faster NK T-cell reconstitution is associated with a reduced incidence of relapse (69, 70).

## Targeting Aging Host for Therapeutic Intervention

Strategies to improve immune reconstitution and HCT outcomes for older patients, we should start with an appropriate selection of younger donors, appropriate transplant platforms, and meticulous supportive care targeting geriatrics related issues peri-transplant. Traditionally, strategies aimed to enhance thymic function have been investigated to enhance post-transplant immune reconstitution, with molecules such as sex hormone ablation, KGF, interleukin-7, growth hormone, IL22, BMP4, RANKL under investigation (10, 44). Recent discoveries in geroscience, however, have stimulated additional interest in exploring therapeutic targets in aging pathways to improve outcomes in cancer therapy.

The contribution of age-related changes in the bone marrow microenvironment on HSC aging has been well studied (71). However, it was not until recently that its therapeutic potential was explored. In an endothelial transplantation experiment, Poulos et al. demonstrated that bone marrow endothelial cells (EC) supported HSC development and infusion of young ECs enhanced hematopoietic recovery following myelosuppressive therapy. Importantly, co-infusion of young ECs augmented aged HSC engraftment and enhanced survival of lethally irradiated mice by mitigating damage to the BM vascular microenvironment (38). These results have significant implications for older patients and immune reconstitution post-HCT. Early phase clinical trials are underway to explore the clinic impact of third party, genetically engineered endothelial cells in mitigating side effects of high dose chemotherapy and to promote HSC recovery post-HCT (NCT03925935).

Senolytics is a group of compounds with selective activity against senescence cells, with the potential to reverse the aging process (72). These compounds are yet to be tested in the setting of human or murine model of allo-HCT or immune reconstitution, although in a landmark study, Chang et al. showed that ABT263, a selective apoptosis inducer of senescence cells in culture when orally administrated to either sublethally irradiated or normally aged mice effectively depleted senescent bone marrow HSCs and mitigated TBI-induced premature aging of the hematopoietic system and rejuvenated the aged HSCs (73, 74). This study suggested that senolytic drugs especially ABT263 may represent a new class of radiation protectors while also stimulate immune reconstitution *via* rejuvenation of HSC post allo-HCT. Other senolytics drugs are in development as well, including some repurposed drugs and even cell therapy using chimeric antigen receptor T-cells (75, 76). One such combination is dasatinib plus quercertin (D+Q) which is clinically safe and has shown potential efficacy in a pulmonary fibrosis model of aging. Kirkland et al. launched the first clinical trial with D+Q to be given to survivors of HCT, a population prone to premature aging (NCT02652052).

Another possible target is SASP and its associated cytokine milieu (72). Notably, some agents which target SASP components are already used for certain disease applications and are potential candidates for drug repurposing. An IL‐1 receptor antagonist, anakinra, an anti‐IL‐6‐receptor antibody, tocilizumab, and tumor necrosis factor‐α inhibitors, such as etanercept and infliximab, are currently used to treat rheumatoid arthritis. As the IL‐1 receptor is a key inducer of many other SASP factors such as IL‐6 and IL‐8, these drugs may be useful for selectively blocking the deleterious impact of SASP (32). Targeted inhibition of the IL‐6 or IL‐8 signaling axis weakens the inflammatory response in murine models of senescence (77).

Another class of agents that could be useful in the allogeneic HCT setting is mTOR inhibitors, which has antiaging and immunomodulatory properties. In a classic murine model of ex vivo HSC expansion, mTOR inhibitor rapamycin promoted long term expansion and hematopoietic reconstitution *via* inhibition of HSC senescence (78, 79). The implication of the study in HCT remains to be explored, as the mTOR inhibitor sirolimus is a commonly used GVHD prevention medication. Even more interestingly, in randomized early-phase trials of older adults, combinatorial inhibition of the mTOR pathway using two synthetic inhibitors was effective in reducing rates of infections and enhancing immune functions as measured by influenza vaccination response and immune regulatory gene upregulation. On the cellular level, mTOR inhibition led to reduced numbers of CD4+ CD8+ T-cells expressing the exhaustion marker program-death 1 receptor (80, 81). Therefore, these agents are attractive compounds to stimulate immune reconstitution post HCT in older adults.

## Conclusion

**Figure 1:**
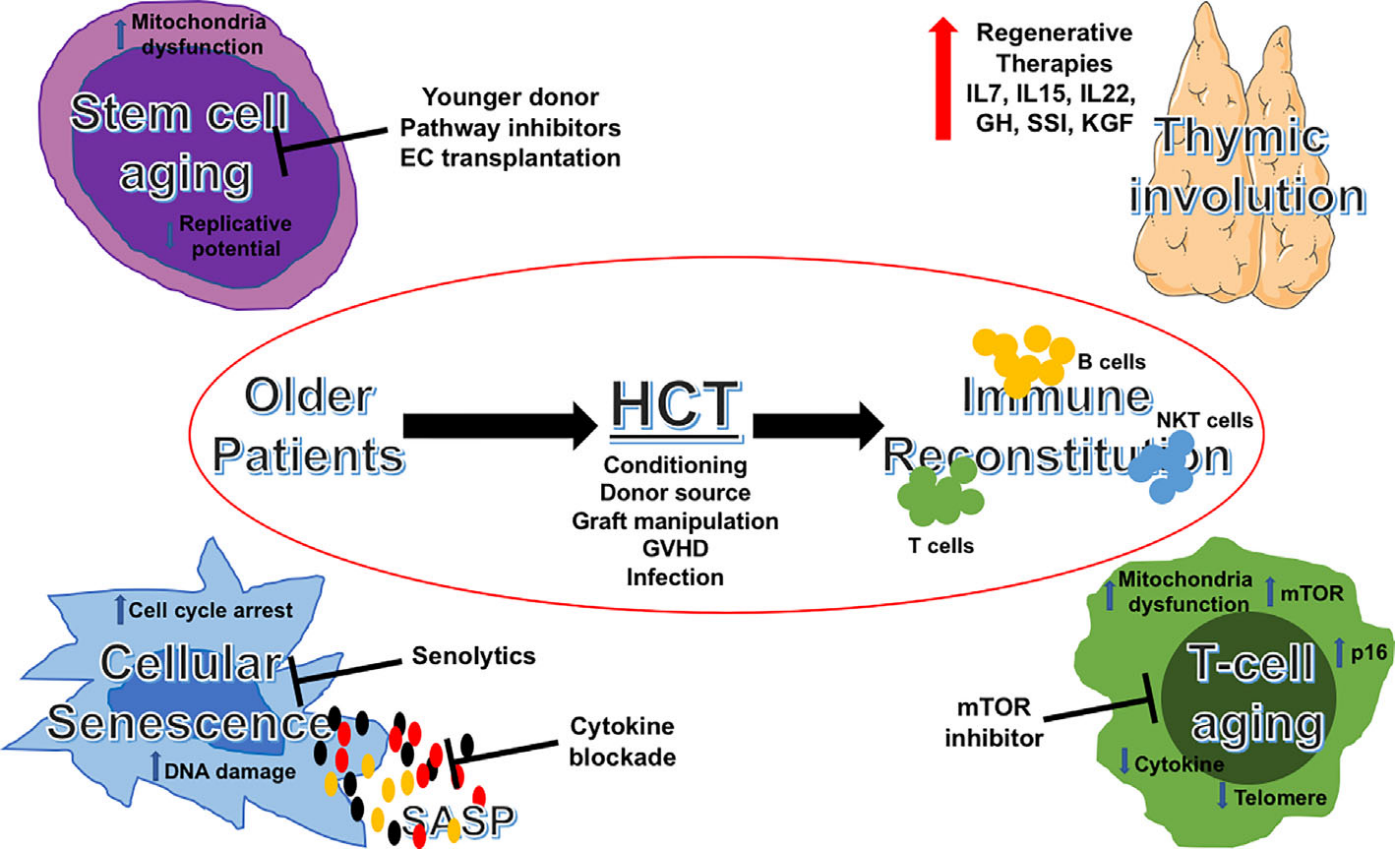
Aging pathways and potential therapeutic targets in allogeneic HCT for older patients. Schematic illustration of potential targetable aging pathways in allogeneic HCT for older patients. HCT, hematopoietic cell transplantation; IL, interleukins; GH, growth hormone; SSI, sex steroid inhibition; KGF, keratinocyte growth factor; GVHD, graft-versus-host disease; SASP, senescence-associated secretory phenotype; mTOR, mechanistic target of rapamycin.

Strategies to improve immune reconstitution and transplant outcomes for older patients could include: an appropriate selection of younger donors, appropriate transplant platforms, and meticulous supportive care targeting geriatrics-related issues peri-transplant. Advances in aging science have provided opportunities to examine their impact on immune reconstitution post-transplant, as well as potential mechanism-based, therapeutic targets to improve outcomes. In **Figure 1**, we summarize these important areas of consideration and their druggable targets. Not only we are learning to better care for the older patient in allo-HCT, but we are also beginning to translate and apply the findings in aging science to improve outcomes of immune recovery post-transplantation. This will hopefully result in better outcomes for older patients undergoing allo-HCT.

## Author Contributions

All authors contributed to the article and approved the submitted version.

## Funding

This research was supported in part by the NIH/NCI Cancer Center Support Grant P30 CA008748 and the Program Project Grant P01 CA023766. The content is solely the responsibility of the authors and does not necessarily represent the official views of the National Institutes of Health.

## Conflict of Interest

MB has received research support from Seres Therapeutics; has consulted, received honorarium from or participated in advisory boards for Seres Therapeutics, Flagship Ventures, Novartis, Evelo, Jazz Pharmaceuticals, Therakos, Amgen, Magenta Therapeutics, WindMIL Therapeutics, Merck & Co, Inc., Acute Leukemia Forum (ALF) and DKMS Medical Council (Board); has IP Licensing with Seres Therapeutics, and Juno Therapeutics. RL has served on the advisory board of Kite – a Gilead Company.

The remaining author declares that the research was conducted in the absence of any commercial or financial relationships that could be construed as a potential conflict of interest.
